# Effects of exogenous bile acids (BAs) on growth, lipid profile, digestive enzymes, and immune responses of thinlip mullet*, Liza ramada*

**DOI:** 10.1038/s41598-023-49788-6

**Published:** 2023-12-18

**Authors:** Mohsen Abdel-Tawwab, Hany M. R. Abdel-Latif, Mohammed F. El Basuini, Asmaa M. El-Nokrashy, Asmaa A. Khaled, Mohamed Kord, Ali A. Soliman, Mohamed Zaki, Abd-Elaziz Nour, Eman M. H. Labib, Hala Saber Khalil

**Affiliations:** 1 Department of Fish Biology and Ecology, Central Laboratory for Aquaculture Research, Agriculture Research Center, Abbassa, Abo-Hammad, Sharqia, Egypt; 2 Department of Poultry and Fish Diseases, Faculty of Veterinary Medicine, Alexandria University, Alexandria, 22758 Egypt; 3 Animal Production Department, Faculty of Agriculture, Tanta University, Tanta, 31527 Egypt; 4 Faculty of Desert Agriculture, King Salman International University, South Sinai, 46618 Egypt; 5 Department of Aquaculture, Faculty of Aquatic and Fisheries Science, Kafrelsheikh University, Kafr Elsheikh, Egypt; 6 Animal and Fish Production Department, Faculty of Agriculture (Saba Basha), Alexandria University, Alexandria, Egypt; 7 Central Laboratory for Agricultural Climate, ARC, Giza, Egypt; 8 National Institute of Oceanography and Fisheries (NIOF), Cairo, Egypt; 9 Animal Production Department, Faculty of Agriculture, Alexandria University, Alexandria, Egypt; 10 Utilization of By-Products Department, Animal Production Research Institute, Agriculture Research Center, Ministry of Agriculture and Land Reclamation, Dokki, Giza, Egypt; 11 Aquaculture Department, Faculty of Fish Resources, Suez University, Suez, 43221 Egypt; 12 College of Fisheries and Aquaculture Technology, Arab Academy for Science, Technology, and Maritime Transport, Alexandria, Egypt

**Keywords:** Developmental biology, Physiology, Zoology

## Abstract

An eight-week trial was designed to explore the dietary effects of commercially purchased exogenous bile acids (BAs) on growth, whole-body composition, lipid profile, intestinal digestive enzymes, liver function enzymes, oxidative stress biomarkers, and serum immunity of thinlip mullet, *Liza ramada*. Four triplicate groups (10.50 ± 0.05 g) were fed four soybean meal (SBM)-based diets supplied with several BAs levels at 0 (control), 50, 130, or 350 mg/kg feed. Results indicated that the growth was significantly increased in groups fed BAs-based diets, especially at 130 mg/kg feed. The body composition analysis showed that feeding fish on diets supplied with BAs up to 130 mg/kg decreased moisture (%) alongside increased crude protein (%). However, the body composition of fish fed a diet with 350 mg BAs/kg had the lowest moisture (%) and the highest crude protein (%). Moreover, there were significant increases in the intestinal (protease, α-amylase, and lipase) enzyme activities in the groups supplied with BAs up to 130 mg BAs/kg. Liver function enzymes (aspartate aminotransferase and alanine aminotransferase enzyme activities) were significantly decreased in BAs-supplemented groups compared to those fed the BAs-free group. On the other hand, the control group had higher total cholesterol, triglycerides, and low-density lipoprotein alongside the lower high-density lipoprotein than BAs-supplemented groups, especially at 350 mg BAs/kg feed. BAs significantly decreased hepatic malondialdehyde concentrations and increased the activity of hepatic catalase, superoxide dismutase, and total antioxidant capacity compared with those reared on the control diet. Serum lysozyme, respiratory burst, and alternative complement activities were significantly increased in BAs-supplied groups, particularly in the group supplied with 130 mg BAs/kg compared to those fed on the control diet. Accordingly, our findings recommend that including 130 mg BAs/kg in an SBM-based diet enhanced the growth, digestive enzyme activities, and liver functions, alleviated oxidative stress, boosted serum immunity, and lowered lipid metabolites in thinlip mullet. These findings will be beneficial for improving the quality of feed prepared for feeding mullets and an effective alternative strategy to support mullet farming.

## Introduction

In the Mediterranean region, there are several mullet species (Mugilidae family), such as thicklip grey mullet (*Chelon labrosus*), thinlip mullet (*Liza ramada*), flathead grey mullet (*Mugil cephalus*), golden grey mullet (*Liza aurata*), and leaping mullet (*Liza saliens*)^1^. Thinlip mullet is characterized by its high commercial value in the markets. It can feed on zooplankton, dead plant matter, and detritus available at the lowest trophic level^2^. In addition, it can consume artificially formulated feeds in different culture systems^3^.

As it is well known, the prices of fishmeal (FM), a primary protein source in aquafeeds, have been substantially increased^4^, mostly due to (a) the extension of aquaculture production with continually increased demand^5,6^ and (b) the shortage of its production^7^. Moreover, In Egypt and several other developing countries, there is another problem of importation of FM from its producers worldwide, because of the decrease of foreign currency^8^. Thus, the aqua-culturists and fish farmers seek to find suitable FM alternatives such as insect meals^9^, mealworms^10^, soybean protein concentrate^11^, high protein distillers dried grains^12^, among others. On the other hand, plant protein sources, including soybean meal (SBM), are commonly utilized to replace FM in aquafeeds as a result of their high availability and low prices paralleled to FM^13^. However, SBM should not be used as a major and single protein source in fish diets since it lacks certain essential nutrients, particularly cholesterol. According to Lin, et al.^14^, the amount of cholesterol in the shrimp hepatopancreas and the hemolymph was reduced linearly as dietary SBM inclusion increased. The impacts of hypocholesterolemia may also be induced by a lack of cholesterol in SBM, and poor cholesterol consumption caused by the presence of anti-nutritional substances such as saponin, phytosterol, and non-starch polysaccharides^15^. Hence, utilizing functional feed additives to improve the quality of aquafeeds, such as antioxidants, pellet binders, and exogenous enzymes, could be considered from the practical ways to mitigate the aforementioned negative effects^16–18^.

Bile acids (BAs) are sterols with amphipathic properties, mainly synthesized in the liver from cholesterol and then released in the intestinal lumen after the intake of diets^19^. BAs are the core components of bile, considered a natural fat emulsifier^20^, and exert a pivotal role in mammalian lipid metabolism^21^. They also help to maintain cholesterol homeostasis in the body^22^. For aquacultural uses, their safety margin makes them a possible supplement in aquafeeds in some fish species^23,24^, until their use is confirmed in a wide range of finfish species after several practical laboratory-based experiments. Previous studies elucidated that optimum BAs supplementation could efficiently enhance productive and hepatic health^25–27^ and lessen the negative impacts of plant protein sources when being added to diets^28^.

Several types of exogenous BAs can be efficiently used in aquafeed, such as chenodeoxycholic acid (CDCA), taurocholic acid sodium, hyocholic acid (HCA), hyodeoxycholic acid (HDCA), and sodium taurolithocholate^29^. With a particular concern, exogenous BAs extracted from pig bile (porcine source) provoked positive effects in the improvement of amino acids metabolic pathways in juvenile European eel livers^30^. Exogenous BAs originated from Ox bile also alleviated hepatitis caused by high dietary starch in *Micropterus salmoides*^31^. Moreover, exogenous BAs comprised of a mixture of taurocholic acid (TCA) and glycocholic acid (GCA) (in a ratio of 2:1) participated in dietary lipid digestion in *Oncorhynchus mykiss*^32^. Of interest, dietary taurocholic acid sodium promoted growth and reduced lipid accumulation in hybrid groupers fed on a high-lipid diet^33^.

Using exogenous BAs containing different levels of HDCA, CDCA, and HCA extracted from various sources have been previously tested in several finfish species with proven efficacy and presented several advantageous uses. Our latterly published paper showed that exogenous BAs (Runeon®) composed of a mixture of HCA, CDCA, and HDCA considerably enhanced the growth and non-specific immunity of *Pangasianodon hypophthalmus*^19^. Although the obtained results, concerns should be taken into account while choosing dietary doses as higher doses can induce hepatoxic effects^34^. According to the benefits of exogenous BAs supplementation as described in the literature, the existing research paper was designed to assess the dietary impacts of various levels of Runeon® on growth, body composition, digestive enzymes, and physiological responses of thinlip mullet-fed a SBM as a plant-based protein.

## Materials and methods

### Ethical statement

The animal study was examined and authorized by the Institutional Animal Care and Use Committee with an Approval Code ALEXU-IACUC: 19/22/10/20/3/25. All methods in this study were performed following the relevant guidelines and regulations and arrive guidelines.

### Bile acids (BAs) and formulation of test diets

Four iso-nitrogenous and iso-lipidic diets were formulated according to the data presented in Table 1. Exogenous BAs powder (RUNEON®; 99% purity) extracted from a porcine source was procured from Shandong Longchang Animal Health Product Co., Ltd. (Shandong, China). In BAs powder, the contents of HCA, HDCA, and CDCA were 8.00%, 70.90%, and 20.20%, respectively (data presented from the manufacturer). The exogenous BAs were supplemented with the basal diet using levels such as 0 (control diet), 50, 130, and 350 mg/kg. The selected BAs doses were mixed thoroughly for 30 min with the diet ingredients. A suitable amount of water was added to each kg feed during the mixing procedures to moisten the diets and get dough. The generated dough was then passed across a meat mincer to form pelletized diets with a 0.5 mm diameter. The formulated test diets were then dried out in the open air (< 10% moisture), then preserved in plastic bags, and kept in a refrigerator (− 4 °C) until utilized.

**Table 1:** Components (g/kg diet) and proximate chemical analysis (% on DM basis) of the control and experimentally formulated diets used for feeding thinlip mullet (*Liza ramada*) in the present study.

Ingredients (g/kg diet)	Diet 1	Diet 2	Diet 3	Diet 4
Soybean meal (46% CP)	460	460	460	460
Meat and bone meal (53% CP)	100	100	100	100
Ground corn (7.5% CP)	100	100	100	100
Wheat bran	160	160	160	160
Rice bran	120	120	120	120
Fish oil	5	5	5	5
Soybean oil	5	5	5	5
Vitamins premix^1^	10	10	10	10
Minerals premix^2^	10	10	10	10
Dicalcium Phosphate	20	20	20	20
Carboxy-methylcellulose	10	9.95	9.87	9.65
Exogenous BAs	0	0.05	0.13	0.35
Total	1000	1000	1000	1000
**Proximate analysis**				
Dry matter	89.65	89.68	89.67	89.69
Crude protein	28.44	28.44	28.44	28.44
Total lipid	5.86	5.82	5.89	5.85
Crude fiber	7.05	6.92	6.96	6.94
Ash	10.24	10.39	10.43	10.41
NFE	48.41	48.43	48.28	48.36
Gross energy (MJ/kg)	17.12	17.11	17.12	17.11

^1^ and ^2^ Vitamin premix and Mineral premix^3^.

Gross energy was expressed based on NRC^35^ as 16.7, 37.4, and 16.7 kJ/*g* for protein, lipid, and carbohydrates, orderly.

### Fish acclimation, husbandry, and rearing conditions

Hatchery-reared thinlip mullets were procured from Gamasa, located near the coastal shoreline of the Mediterranean Sea, Daqahliya Province, Egypt. Fish were then transferred to the National Institute of Oceanography and Fisheries at Baltim Research Station, Egypt. All fish were left in four cement raceways for one month to be acclimated to the new rearing conditions and to allow them to feed on a formulated pelleted diet. The long acclimation period was selected because mullet species usually refuse commercially formulated pelleted diets during acclimation once they are captured from the wild^36^. During the adaptation period, fish were offered a pelleted commercial tilapia diet (35% crude protein). Fish were then stocked with a mean initial weight of 10.50 ± 0.05 g (means ± S.E.) into 12 hapas with dimensions (0.7 m × 0.7 m × 1.0 m) that were fixed in the raceway ponds. To maintain adequate aeration, all hapas were kept with fish (10 fish/hapa) and were supplied by two air stones linked with blower motors. For eight weeks, fish were fed on the relevant test diets three times per day (8.00 am, 2.00 pm, and 8.00 pm) until full satiety. Water quality parameters were regularly monitored at weekly intervals. The water parameters as dissolved oxygen, temperature, pH, nitrite, salinity, and unionized ammonia values in all hapas were kept at 6.6 ± 0.3 mg/L, 27.0 ± 1.5 °C, 7.5 ± 0.5, 0.03 ± 0.01 mg/L, 7.0‰, and 0.03 ± 0.01 mg/L, respectively. All these parameters were within the suitable range for fish rearing.

### Growth performance, feed utilization, and somatic indices

At the end of the feeding trial (8 weeks), fish from each hapa were netted, gathered, counted, and group-weighed. Following that, the fish were aseptically necropsied, and the viscera and liver were removed, and weighed, and somatic indices were assessed. The Fulton condition (K) factor, viscera somatic index (VSI), and hepato-somatic index (HSI) were also assessed. Equations used for evaluation of the growth and feed utilization indices such as weight gain percentage (WG%), specific growth ratio (SGR), feed intake (FI), feed conversion ratio (FCR), and fish survival (%) have been previously published in our recent paper^37^.

WG % = 100 × (Wt60 – Wt0) / Wt0.

SGR (%/day) = 100 [Ln Wt60—Ln Wt0] / 60.

Feed intake (FI; g feed per fish) = total feed consumed/fish number per hapa.

FCR = FI (g)/ WG (g).

Fish survival (SR %) = 100 [Number of fish after 60 days/ Number of fish at the start].

K factor (%) = 100 × body weight (g)/ [body length (cm)]^3^.

VSI (%) = 100 [viscera weight (g) /body weight (g)].

HSI (%) = 100 [liver weight (g)/body weight (g)].

### Proximate chemical analysis

According to protocols described by the Association of Official Analytical Chemists^38^, the proximate chemical contents of diets and whole fish body were assessed. After 105 °C oven drying up to a constant dry weight, moisture content was assessed. A Kjettech autoanalyzer (Model 1030 Tecator Hoganas Sweden) was utilized to assess crude protein content, and a Soxtec extractor (Lab-Line Instruments, Inc., Melrose Park, Illinois, USA) was used to determine the total lipid content. After samples were burned at 550 °C for six hours in a muffle furnace (Thermolyne Corporation, Dubuque, Iowa, USA), the weight loss was used to quantify the amount of ash (%).

### Blood and tissues sampling

Fish in all hapas were starved for one day following the feeding trial to collect blood and tissue samples. Before autopsy, fish were anesthetized using clove oil (5 mg/L) and blood samples from caudal veins were taken from fish in each hapa (10 fish/group) and divided between two sets of Eppendorf tubes. One set of samples was collected with sodium heparin (20 U/L) as an anticoagulant and used for measuring white blood cell (WBC) count, red blood cell (RBC) count, hemoglobin (Hb), and hematocrit (Hct %). The second set was collected without anticoagulant and centrifugated at 5000 × *g* for 20 min to collect serum to assess serum biochemical indices. Fish were dissected after blood samples, and the mid-intestine and liver were sampled under aseptic conditions. They were washed and promptly homogenized in a physiologically normal saline solution. After that, the tubes were centrifugated at 10,000 × *g* for 10 min in a cooling centrifuge (Thermo Scientific) at 4 °C. The supernatant was collected in test tubes and kept at − 20 °C until usage.

### Haemato-biochemical analysis

RBC and WBC counts were manually determined using Hayek’s solution diluent and a Neubauer's hemocytometer^39^. Based on van Kampen and Zijlstra^40^, thecyanomethaemoglobin technique was used to determine the Hb concentrations. To determine the Hct%, fresh blood was deposited in capillary glass tubes immediately after sampling and spun for 10 min in a micro-hematocrit centrifuge. Next, the packed cell volume was determined. The mean corpuscular volume (MCV), mean corpuscular hemoglobin (MCH), and mean corpuscular hemoglobin concentration (MCHC) were assayed according to^41^. Serum biochemical indices such as alanine aminotransferase (ALT), aspartate aminotransferase (AST), total cholesterol (T-CHO), triglycerides (TG), low-density lipoprotein cholesterol (LDL-c), and high-density lipoprotein cholesterol (HDL-c) were measured via using diagnostic kits (Spinreact, S.A., Gerona, Spain).

### Digestive enzyme activities

Using commercial diagnostic kits (Cusabio Biotech Co. Ltd., Wuhan, Hubei, China), digestive enzyme activities were assessed in the middle intestine samples per manufacturer recommendations. The α-amylase enzyme activities were assessed in accordance with the methodology explained by Bernfeld^42^. The protease enzyme activities were estimated by the casein digestion method previously described by^43^. The lipase enzyme activities were assayed according to the method of^44^. The levels of α-amylase, protease, and lipase enzyme activities in the fish intestinal samples were assessed as Units per mg/protein.

### Liver antioxidant variables and immunological assays

Hepatic catalase (CAT) and superoxide dismutase (SOD) were measured using protocols described in^45,46^, with the aid of diagnostic kits (MyBioSource Inc., San Diego, California, USA) according to the producer’s instructions. The total antioxidant capacity (TAC) of the hepatic specimens was also estimated in harmony with the ferric reduction antioxidant power (FRAP) assay described by Benzie and Strain^47^ at an optical density OD of 593 nm. Malondialdehyde (MDA) contents were evaluated (as lipid peroxidation indicator) in hepatic tissues at an OD of 532 nm by thiobarbituric acid substances (TBARS) according to the assay previously described^48,49^. According to Ellis^50^, serum lysozyme activity (LYZ) was assayed as the amount of the enzyme, which led to a decline in absorbance of 0.001 min/1 mL serum. The nitro blue tetrazolium (NBT) test (Sigma-Aldrich, USA) was used to evaluate the respiratory burst activity (RBA), which was then quantified via a spectrophotometer at OD = 540 nm^51^. According to the Yano^52^ technique, alternative complement activity (ACH50; U/mL) in which the serum concentration that causes 50% hemolysis was determined in each group.

### Statistical analysis

The data were examined for the normality of distribution and the homogeneity of variances among BAs-supplemented groups by Kolmogorov–Smirnov and Bartlett's tests, respectively. Data were investigated by one-way ANOVA to evaluate BAs effects on the different measurements. Differences among means were statistically significant at *P* < 0.05. Analysis was performed by the SPSS program and GraphPad Prism X8.

## Results

### Effects of BAs on growth, somatic indices, and feed utilization

**Figure 1:**
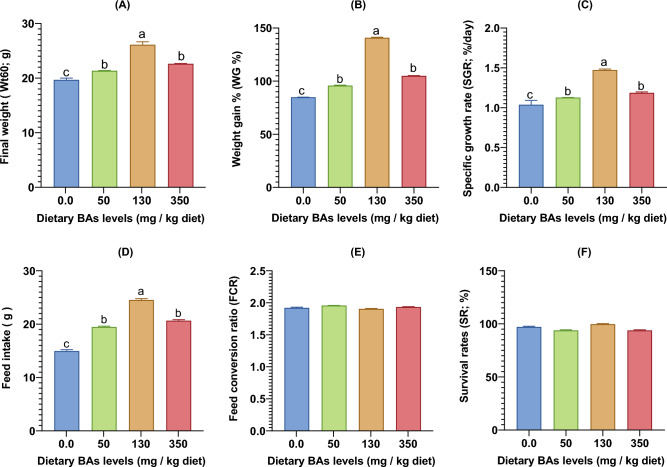
Final weight (**A**), weight gain % (**B**) specific growth rate (**C**), feed intake (**D**), feed conversion ratio (**E**), and survival rates (**F**) of thinlip mullet (*Liza ramada*) fed on diets supplied with different levels of bile acids (BAs) for 8 weeks. Data were expressed as Means ± S.E. Different letters of each chart indicate significant differences among different groups at *P* < 0.05.

The highest growth indices, including Wt60 (Fig. 1A), WG% (Fig. 1B), SGR (Fig. 1C), and FI (Fig. 1D), were noted in BAs-supplied fish, especially in the groups fed on diets with 130 mg/kg feed compared with those fed control diet. Oppositely, non-significant differences (P > 0.05) were noted in FCR values (Fig. 1E) and survival rates (%) (Fig. 1F) among the test groups. In the same sense, no significant differences (P > 0.05) were also detected in the biometric indices including K factor, HSI, and VSI (Table 2), among the experimental groups.

**Table 2:** Condition factor, somatic indices, and proximate chemical composition (% on a fresh weight basis) of the whole-body of thinlip mullet fed on diets supplied with different levels of bile acids (BAs) for 8 weeks.

Parameters	BAs levels (mg/kg diet)				P value
	0.0 (control)	50	130	350	
**Body indices**					
Condition factor (CF)	0.74 ± 0.010	0.76 ± 0.029	0.73 ± 0.029	0.75 ± 0.038	0.934
Hepatic-somatic index (HSI)	1.05 ± 0.086	1.21 ± 0.039	1.27 ± 0.074	1.19 ± 0.056	0.179
Viscera-somatic index (VSI)	10.0 ± 0.20	10.5 ± 0.52	9.8 ± 0.33	10.8 ± 0.32	0.220
**Proximate chemical composition**					
Moisture (%)	66.2 ± 201 a	66.4 ± 0.30 a	66.2 ± 0.29 a	64.5 ± 0.37 b	0.001
Crude protein (%)	15.9 ± 0.38 b	16.0 ± 0.13 b	15.9 ± 0.13 b	18.4 ± 0.19 a	0.004
Crude lipids (%)	7.6 ± 0.06	7.5 ± 0.12	7.6 ± 0.25	7.3 ± 0.15	0.085
Ash (%)	4.7 ± 0.09	4.7 ± 0.08	4.8 ± 0.08	4.7 ± 0.20	0.829

Means with the same letter in the same row are not significantly different at *P* < 0.05.

### Effects of BAs on the whole-body composition

The proximate chemical composition of whole-body of thinlip mullet (Table 2) presented that fish fed on a diet supplied with 350 mg BAs/kg showed the lowest moisture content (64.5%) accompanied by the highest protein content (18.4%). However, non-significant differences (P > 0.05) were perceived in the contents of lipids (%) and ash (%) in the whole body of fish fed on diets supplied with different BAs levels, and their ranges were 7.3% – 7.6% and 4.7% – 4.8%, respectively (Table 2).

### Effects of BAs on hematological indices

It was noticed that the counts of WBCs and RBCs alongside the Hb concentrations and Hct (%) levels were significantly increased (*P* < 0.05) as dietary BAs levels increased, reaching their utmost levels in the group fed a diet supplied with 130 mg BAs/kg (Table 3). The highest MCV values were noted in group fed on the control and 350 mg BAs/kg diets. The lowest MCH was found in the fish group fed on a diet supplied with 50 mg BAs/kg feed with non-significant differences (P > 0.05) among other BAs treatments. Moreover, it was found that feeding mullets on a diet supplied with 130 mg BAs/kg revealed the highest MCHC values, while other BAs levels showed non-significant differences among them (P > 0.05).

**Table 3:** Hematological parameters and serum metabolites of thinlip mullet fed on diets supplied with different levels of bile acids (BAs) for 8 weeks.

Parameters	BAs levels (mg/kg diet)				P value
	0.0 (control)	50	130	350	
**Hematological parameters**					
WBCs (× 10^3^/mm^3^)	19.9 ± 0.91 c	24.2 ± 0.89 b	28.6 ± 0.23 a	23.7 ± 0.69 b	0.008
RBCs (× 10^6^/mm^3^)	3.80 ± 0.232 c	4.54 ± 0.131 b	5.02 ± 0.219 a	4.28 ± 0.117 b	< 0.001
Hb (g/dL)	11.6 ± 0.35 c	12.6 ± 0.38 b	14.6 ± 0.40 a	12.2 ± 0.35 bc	< 0.001
Hct (%)	48.8 ± 2.47 c	53.5 ± 1.50 ab	56.5 ± 1.50 a	52.2 ± 2.93 bc	0.017
MCV (μm^3^/cell)	128.4 ± 3.91 a	117.8 ± 2.00 bc	112.5 ± 2.32 c	122.0 ± 10.03 ab	0.034
MCH (pg/cell)	30.5 ± 1.64 a	27.8 ± 1.09 b	29.1 ± 0.59 ab	28.5 ± 0.92 ab	0.027
MCHC (%)	23.8 ± 0.75 b	23.6 ± 0.58 b	25.8 ± 0.95 a	23.4 ± 1.33 b	0.017
**Serum metabolites**					
T-CHO (mg/dL)	284.8 ± 0.42 a	257.4 ± 1.87 b	194.3 ± 0.39 c	177.4 ± 0.49 d	< 0.001
TG (mg/dL)	257.7 ± 1.33 a	212.6 ± 1.26 b	197.5 ± 0.42 c	145.8 ± 0.87 d	< 0.001
LDL-c (mg/dL)	216.3 ± 1.29 a	196.1 ± 1.38 b	149.0 ± 2.17 c	132.0 ± 0.79 d	< 0.001
HDL-c (mg/dL)	20.5 ± 0.82 c	27.2 ± 0.97 b	28.4 ± 0.68 b	34.1 ± 0.77 a	< 0.001

Means with the same letter in the same row are not significantly different at *P* < 0.05.

Hb: Hemoglobin; Hct: Hematocrit; HDL-c: High-density lipoprotein cholesterol; LDL-c: Low-density lipoprotein cholesterol; MCH: Mean corpuscular hemoglobin; MCHC: Mean corpuscular hemoglobin concentration; MCV: Mean corpuscular volume; RBCs: Red blood cells; T-CHO: Total cholesterol; TG: Triglycerides; WBCs: White blood cells.

### Effects of BAs on lipid profile

Regarding the lipid profile, it was noticed that there were significant decreases (*P* < 0.05) in T-CHO, TG, and LDL-c values in mullet as BAs inclusion levels increased, reaching their lowest levels in the treatment of 350 mg/kg feed (Table 3). Conversely, significant increases (*P* < 0.05) in HDL-c levels were noticed and associated with the rise in dietary BAs levels up to 350 mg/kg feed (Table 3).

### Effects of BAs on liver functions

**Figure 2:**
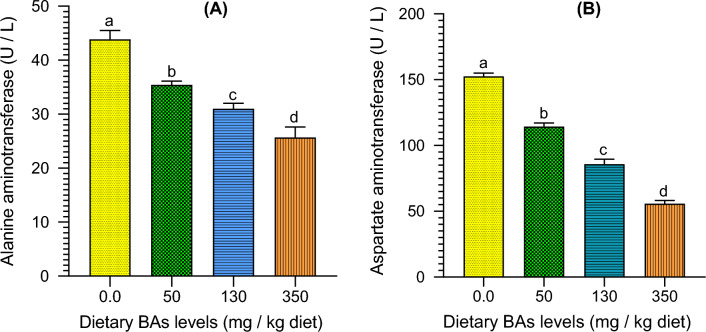
The liver function enzymes including (**A**) alanine aminotransferase (ALT; U/L) and (**B**) aspartate aminotransferase (AST; U/L) enzyme activities of thinlip mullet (*Liza ramada*) fed on diets supplied with different levels of bile acids (BAs) for 8 weeks. Data were expressed as Means ± S.E. (n = 5). Different letters of each chart indicate significant differences among different groups at *P* < 0.05.

There was an opposite correlation between the dietary BAs levels and the values of liver function enzymes (ALT and AST), as their levels were significantly (*P* < 0.05) decreased in mullet as BAs levels increased, and their lowest levels were found in the group fed on a diet supplied with 350 mg/kg feed (Fig. 2).

### Effects of BAs on intestinal digestive enzymes

**Figure 3:**
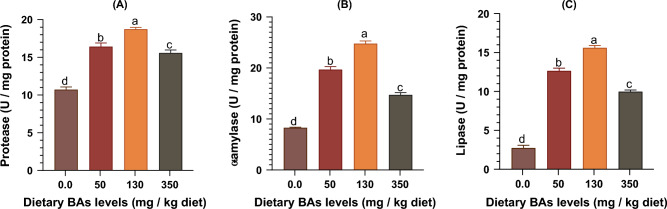
The intestinal digestive enzyme activities including (**A**) proteases, (**B**) α-amylase, and (**C**) lipase enzyme activities of thinlip mullet (*Liza ramada*) fed on diets supplied with different levels of bile acids (BAs) for 8 weeks. Data were expressed as Means ± S.E. (n = 5). Different letters of each chart indicate significant differences among different groups at *P* < 0.05.

The intestinal protease, α-amylase, and lipase enzyme activities were significantly (*P* < 0.05) enhanced as BAs levels increased in diets up to 130 mg/kg feed and after which the activities of these enzymes decreased (Fig. 3).

### Effects of BAs on hepatic antioxidants variables

**Figure 4:**
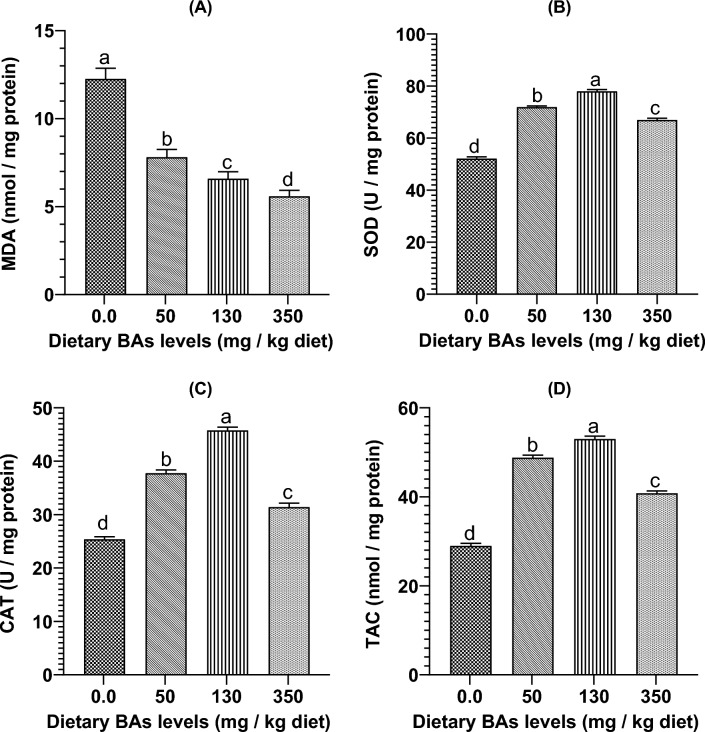
Hepatic oxidative stress biomarkers including (**A**) malondialdehyde (MDA), (**B**) superoxide dismutase (SOD) enzyme, (**C**) catalase (CAT) enzyme, and (**D**) total antioxidant capacity (TAC) of thinlip mullet (*Liza ramada*) fed on diets supplied with different levels of bile acids (BAs) for 8 weeks. Data were expressed as Means ± S.E. (n = 5). Different letters of each chart indicate significant differences among different groups at *P* < 0.05.

In relationship to the group fed BAs-free diet, significant decreases (*P* < 0.05) in hepatic MDA concentrations alongside significant increases in hepatic CAT, SOD, and TAC levels (*P* < 0.05) were noted in fish groups fed on BAs-supplied diets (Fig. 4).

### Effects of BAs on serum immune parameters

**Figure 5:**
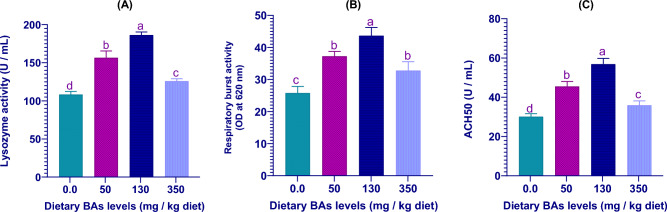
The serum immune parameters including (**A**) lysozyme activity, (**B**) respiratory burst activity, and (**C**) alternative complement activity (ACH50) of thinlip mullet (*Liza ramada*) fed on diets supplied with different levels of bile acids (BAs) for 8 weeks. Data were expressed as Means ± S.E. (n = 5). Different letters of each chart indicate significant differences among different groups at *P* < 0.05.

Feeding mullet on BAs-enriched diets showed a gradual increase of the serum immune parameters (LYZ, RBA, and ACH50 levels) as dietary BAs increased and reached their highest levels in the fish group fed on a diet supplied with 130 mg BAs/kg feed and after which the of the above-noted variables declined (Fig. 5). Moreover, the lowest values of immune biomarkers were detected in fish fed on the control diet (Fig. 5).

## Discussion

We assessed the dietary impacts of exogenous BAs (Runeon®) on the growth and overall performances of thinlip mullet fed for eight weeks on SBM-based diets. The findings showed that mullets fed on SBM-based diets with no exogenous BAs had a depressed growth rate compared to BAs-supplemented groups. Romarheim, et al.^53^ declared that *O. mykiss* fed on diets higher in soybean white flakes or toasted SBM resulted in a considerably decreased BAs content in the digesta of both the proximal and distal intestine than those fed with an FM-based diet. The authors declared that their findings might be linked with the anti-nutritional factors such as soy saponins or isoflavones, which may interact with bile in several ways^53^. It is well-known taurine or cholesterol is required for endogenous BAs biosynthesis^54^. Moreover, several plant protein sources, such as SBM, are deficient in cholesterol^55^. Thus, the deficiency (or decreased amounts) of taurine or cholesterol in plant ingredients may lead to the inadequate synthesis of bile in fish fed principally on plant-based diets. Consequently, this will lead to poor lipid utilization and growth retardation^28^. Another explanation of the growth retardation in the group fed solely on an SBM-based diet may be linked with SBM-induced enteritis, which may negatively impact nutrient digestion and absorption and consequently result in growth retardation^56,57^.

We found that mullet growth was significantly enhanced when fed for eight weeks on diets enriched with exogenous BAs, and their highest growth rates were found in the group-fed diet supplied with 130 mg BAs/kg compared to other groups. Several reports showed that exogenous BAs supplementation to plant protein-based diets could be beneficial in enhancing the growth, as in the case of *O. mykiss* such as 1% cholyltaurine^58^ and 1.5% bovine bile salts^59^. Similar findings were also reported in turbot, which fed a diet with a high level of plant protein and supplied with 0.5% taurocholate^60^. In addition, it was found that dietary supplementation with exogenous BAs containing HCA, HDCA, and CDCA up to 0.15 g/kg diet promoted the growth of GIFT tilapia that fed on a high-plant protein diet^28^. Several mechanisms of action have been published describing the possible reasons for the enhanced growth rates of fish-fed BAs-supplied diets. For example, reports showed that exogenous bovine bile salts might help maintain the integrity of the fish intestinal epithelium, as previously described by Yamamoto, et al.^59^, who found that the intestinal lesions occurred in *O. mykiss*-fed SBM-based diets were not evident when 1.5% exogenous BAs was supplied as a dietary supplement. Furthermore, Yamamoto, et al.^61^ elucidated that supplementing SBM-based diets with 1.5% bovine bile salts or 1.0% sodium cholyltaurine prevented the development of SBM-stimulated intestinal lesions in *O. mykiss*.

Exogenous BAs could also enhance the intestinal digestive ability of fish, as seen in our study, which will lead to enhanced digestibility of nutrients. Zhou, et al.^25^ informed that the growth of grass carp had been promoted in groups fed on diets supplied with exogenous BAs due to increased lipase enzyme activities which led to enhanced lipid digestion and absorption. Yin, et al.^27^ also declared that the growth-stimulating roles of CDCA might be linked to enhanced intestinal digestive enzyme activities and improved lipid utilization in *M. salmoides* juveniles. Dietary exogenous BAs containing HCA, HDCA, and CDCA could also maintain normal hepatic histomorphology and enhance the glucose and lipid metabolism of fish, as in the case of *M. salmoides* fed with a high starch diet^23^. Although the theories mentioned above, the accurate modes of action of exogenous BAs in the fish growth enhancement warrant additional investigations. Moreover, to establish a relationship between the intermediary energetic metabolism of the liver with the activities of digestive enzymes and growth, it would be necessary to have measured the level of metabolites of the liver and of the plasma, such as the rate of free amino acids, triglycerides, fatty acids, glucose, glycogen, lactate, pyruvate, ammonia, and protein. These parameters would necessitate additional research studies.

Regarding the palatability of BAs-supplemented diets, it was found that exogenous BAs could enhance the feed palatability up to 130 mg BAs/kg diet, which manifested herein, by the increased feed consumption in comparison with those fed on the control diet. Hence, dietary supplementation with exogenous BAs could be suitable for only up to 130 mg/kg diet for mullets. On another side, it was found that the high dietary levels of BAs (350 mg/kg feed) in the present study depressed the growth of mullets, and these findings may be linked to the reduced feed intake (FI) in this group. Interestingly, the reduction in FI may occur due to higher BAs levels may reduce the diet palatability and hence will reduce the consumption of feeds. Jiang, et al.^28^ also declared that excess dietary BAs (a mixture of HCA, HDCA, and CDCA) resulted in gallstone formation, disturbed lipid metabolism, and decreased growth performance of GIFT tilapia. Dissimilar results were reported by Ding, et al.^26^, who indicated that growth was promoted with supplementation in *Larimichthys crocea* juveniles fed high-lipid diets supplied with commercial BAs ranging from 300 mg/kg to 450 mg/kg. As observed above, these inconsistencies among the published studies may be coupled with differences in fish species, diet composition, and BAs supplementation doses.

The impacts of exogenous BAs on the whole-body composition of thinlip mullet herein showed lower moisture content and higher crude protein content at the treatment of 350 mg BAs/kg feed only, but no changes in lipids and ash contents were noticed. The study conducted by Zeng, et al.^62^ noticed that exogenous BAs significantly increased crude protein content but did not impact moisture and ash content in the body of juvenile *Schizothorax prenanti*. According to Sun, et al.^63^, dietary BAs significantly reduced moisture, increased crude protein, and lowered ash in the body, muscles, and liver of turbot. However, Gu, et al.^60^ illustrated that dietary taurocholate did not impact moisture, crude protein, or ash contents in juvenile turbot. In the study of Jiang, et al.^28^, exogenous BAs had non-significant impacts on the moisture content in the whole-fish body but had lower muscle crude protein and lipid deposition in the GIFT tilapia body. Dietary 1.5% bovine bile salts noticeably reduced protein digestibility in *O. mykiss* but did not impact the overall body protein levels^59^. In other studies, dietary taurocholate did not significantly influence the whole-body lipid content of turbot^60^. Exogenous BAs supplementation also did not affect the whole-body lipid content in bullfrog (*Rana catabo*)^64^. These contradictory findings may be due to various factors, such as the fish species, the type of feed ingredients used, or other cultural conditions^34^.

Herein exogenous BAs up to 130 mg/kg feed enhanced the intestinal protease, α-amylase, and lipase activities of thinlip mullet. These results suggest that exogenous BAs could enhance carbohydrate, lipids, and protein digestion in this fish species. This evidence could support the roles of BAs in growth stimulation that occurred in this experiment, as we described before. In the present study, the activities of the intestinal digestive enzymes decreased in the group-fed diet supplied with 350 mg BAs/kg diet. Our findings differ from those reported by Zeng, et al.^65^, who found that the highest activities of intestinal lipase were found in *Schizothorax prenanti* juveniles fed with a diet containing 300 mg BAs/kg diet. The intestinal digestive ability of the tongue sole was improved by increased lipase activities in the group supplied with 900 mg BAs/kg diet and increased amylase activity in the groups supplied with 300 mg/kg and 900 mg/kg^66^. Recently, it was found that the activities of hepatic protease, lipase, and amylase enzymes were increased along with the increase of dietary BAs levels (300 and 900 mg/kg) in diets of tongue sole^67^.

In aquaculture, studying hematological indices is considered an imperative tool for examining the health and nutritional status of fish^68,69^. Herein WBCs, RBCs, Hb levels, and Htc % significantly increased as dietary BAs increased and reached their highest values in the group of 130 mg BAs/kg feed. A previous study found that the dietary application of exogenous BAs in a dose rate of 350 mg/kg significantly increased the leukocyte count in *M. salmoides* juveniles fed a high starch-based diet^70^. However, the precise mechanism of improvement of the fish hematological parameters of thinlip mullet in the present study is unclear and warrants further investigation. Although no previous studies were published on the effects of exogenous BAs supplementation on fish hematology, we propose that the improvement of the hematological parameters may be coupled with the improvement of feed utilization and growth performance of BAs-supplied fish groups.

Regarding the influences of exogenous BAs on the lipid profile of thinlip mullet, it was found that T-CHO, LDL-c, and TG significantly decreased as BAs inclusion levels increased. In mammals, it is well-known that processes of regulation of cholesterol homeostasis in the body are affected by several factors, including the absorption of cholesterol from exogenous sources, the biosynthesis of endogenous cholesterol, and the excretion of cholesterol through BAs in fecal matter^71^. Cholesterol is transported in serum when combined with lipoproteins to form LDL-c and HDL-c^72^. The present study provided no exogenous cholesterol to fish in the formulated diets. Therefore, the measured cholesterol (T-CHO, LDL-c, and HDL-c) in the fish serum came from endogenous biosynthesis. The decreased contents of the measured lipid parameters may be associated with the roles of exogenous BAs supplementation, which could promote the activities of lipoproteins and upregulate the LDL receptors and very low-density lipoprotein (VLDL) receptor, consequently, will lead to a decrease in the plasma TG contents^73^. However, explaining the roles of exogenous BAs on the lipid metabolism requires further studies of the molecular mechanisms.

Regarding the effects of exogenous BAs supplementation on the lipid profile of fish, many inconsistent results were found in the previously published literature among finfish species. An earlier study showed that dietary bovine bile salts supplementation decreased the plasma cholesterol content in *O. mykiss*^59^. Also, serum T-CHO decreased in cobia (*Rachycentron canadum*) fed on a BAs-supplemented diet^74^. Dietary exogenous BAs enhanced the overall T-CHO, LDL-c, and HDL-c values in GIFT tilapia^28^. Dietary BAs supplementation significantly decreased serum T-CHO and HDL-c levels with no significant impacts on TG and LDL-c in grass carp^25^. Moreover, it was found that dietary supplementation with 0.02% BAs containing a mixture of HCA, HDCA, and CDCA significantly decreased TG, T-CHO, and LDL-c values in tiger puffer-fed normal and high-lipid diets^75^. Moreover, dietary CDCA effectively decreased TG contents in the plasma of *M. salmoides* juveniles fed a high-fat diet^27^. Likewise, dietary supplemental taurocholic acid sodium also decreased T-CHO, LDL-c, and TG in the serum of hybrid groupers fed a high-lipid diet^33^. Differently, it was reported that dietary BAs significantly increased TG and T-CHO in the plasma of *M. salmoides* juveniles that were fed a high starch-based diet^70^. Notably, the inconsistencies found among the published studies, as mentioned above, may be associated with different variables, such as supplemental doses of exogenous BAs, types of BAs used in diet formulation, and diet ingredients used as basal constituents (lipid-, carbohydrate-, or protein-based diets).

ALT and AST enzymes are the key bioindicators for assessing liver functions and reflecting the hepatic status of the fish^37^. The increased activities of these enzymes are regarded as indicators of degenerative changes and hepatic injury^76^. The present study showed that liver function enzymes (ALT and AST) in mullets significantly decreased as dietary BAs increased, and their lowest levels were found in groups fed a diet containing 350 mg BAs/kg. Moreover, the highest levels were noticed in the control group fed an SBM-based diet. Therefore, it is worthily noted that these findings could demonstrate the potential role of dietary BA to alleviate the negative effects of a plant protein-based diet on liver functions. The plasma ALT enzyme activities decreased, and plasma AST increased significantly in *M. salmoides* fed a high starch diet supplied with exogenous BAs as a dietary supplement^23^. However, ALT and AST enzyme activities in the plasma of *M. salmoides* were significantly decreased when exogenous BAs (350 mg/kg diet) were supplied to a high-starch diet^70^. There is a decreasing trend in the levels of plasma ALT and AST activities in *Cyprinus carpio* fed a high plant protein diet supplemented with exogenous BAs containing a mixture of HCA, HDCA, and CDCA (60 or 600 mg/kg diet)^77^. Latterly, it was noted that hepatic ALT and AST significantly decreased when exogenous BAs were supplemented in diets of tongue sole^67^. Nonetheless, dietary BAs did not significantly affect serum ALT and AST enzyme activities of grass carp^25^. These disagreements may be associated with fish species differences, dietary BAs doses, types, or diet ingredients.

MDA is the end product of lipid peroxidation and is considered a secondary product of oxidative stress^78^. SOD and CAT are classical antioxidant enzymes that protect host cells and tissues from oxidative stress injury^18^. The present study showed significant decreases in hepatic MDA concentrations and increases in hepatic SOD, CAT, and TAC values in mullet-fed SBM-based diets and supplied with various levels of exogenous BAs. The findings suggested beneficial antioxidant effects of exogenous BAs in the treated fish. The enhanced antioxidation capabilities of the treated mullets may be associated with improved growth performance and enhanced liver functions. Our findings were in concordance with several previously published literature. For example, it was found that the hepatic MDA concentrations were decreased along with higher SOD, CAT, and TAC activities in the liver of *L. crocea* juveniles fed high lipid-based diets supplemented with exogenous BAs^26^. Also, similar findings were reported in yellow croaker juveniles fed a high starch-based diet supplemented with exogenous BAs (350 mg/kg diet) for eight weeks^70^. The study by Li, et al.^66^ declared that dietary BAs significantly enhanced the intestinal antioxidant activities of tongue sole by increasing the intestinal SOD and CAT enzyme activities and decreasing the intestinal MDA contents levels. Hepatic MDA contents were significantly decreased, and hepatic SOD activities were significantly increased in *M. salmoides* juveniles fed a diet containing increasing levels of exogenous BAs^27^.

LYZ, RBA, and ACH50 play chief roles in fish immunity^17,79,80^.The present study found that feeding thinlip mullet on BAs-enriched diets significantly boosted the serum immune biomarkers as LYZ, RBA, and ACH50 levels, and their highest levels were detected in the fish group fed on diets supplied with 130 mg BAs/kg feed. The lowest levels of immune biomarkers were detected in fish fed on an SBM-based diet with no BAs supplementation. In coherence with the current findings, noticeable alterations in the immune responses of many fish species after they feed on diets with high plant protein presence amounts such as *Solea solea*^11^. Additionally, Sitjà-Bobadilla, et al.^81^ found that the immunological defense mechanisms of *Sparus aurata* were reduced when fed on diets composed mainly of plant protein components. On the other hand, exogenous BAs also enhanced the immune parameters as LYZ activities and immunoglobulin M contents in tongue sole^66^ and *M. salmoides* juveniles^70^. Until now, little information about the exact immunomodulatory mechanisms of dietary exogenous BAs in fish was known, and thus, additional research is still necessitated.

## Conclusions

The current research outcomes suggested that dietary supplementation with exogenous BAs (Runeon®) in a dose of 130 mg /kg to an SBM-based diet could be suitable to improve the growth and physiological responses of thinlip mullets. Exogenous BAs also alleviated oxidative stress, boosted serum immunity, improved liver functions and digestive enzyme activities, as well as decreased lipid metabolites of thinlip mullet. These findings suggest that dietary BA could improve the functionality of aquafeed prepared for mullet farming. They also propose a novel practical interference for mitigating the negative impacts of using plant protein sources as SBM in fish diets with a possible cost-effective approach to be included in the aqua-feed industry.

## Data Availability

Data is available from the corresponding author upon reasonable request.
